# Different levels of let-7d expression modulate response of FaDu cells to irradiation and chemotherapeutics

**DOI:** 10.1371/journal.pone.0180265

**Published:** 2017-06-30

**Authors:** Katarzyna Monika Lamperska, Tomasz Kolenda, Anna Teresiak, Anna Kowalik, Marta Kruszyna-Mochalska, Weronika Jackowiak, Renata Bliźniak, Weronika Przybyła, Marta Kapałczyńska, Piotr Kozlowski

**Affiliations:** 1 Cancer Genetic Laboratory, Greater Poland Cancer Centre, Poznan, Poland; 2 Postgraduate School of Molecular Medicine, Medical University of Warsaw, Poland; 3 Medical Physics Department, Greater Poland Cancer Centre, Poznan, Poland; 4 Pediatric Research, Division of Pediatric and Adolescent Medicine, Oslo University Hospital Rikshospitalet, Oslo, Norway; 5 Charite University Medicine Berlin, Department of Gastroenterology and Hepatology, Max Planck Institute for Infection Biology, Berlin, Germany; 6 Institute of Bioorganic Chemistry, Polish Academy of Sciences, Poznan, Poland; University of South Alabama Mitchell Cancer Institute, UNITED STATES

## Abstract

The implication of the let-7 family in cancer development is multifaceted. The family acts as tumor suppressor miRNA although overexpression of let-7 has also been described in many types of cancer, including head and neck squamous cell carcinoma (HNSCC). The aim of this study includes whether different expression levels of let-7d has an influence on chemo- and radiosensitivity. FaDu cell line models with a gradually increased level of let-7d (models from A to E) were generated with the lentiviral system. Expression levels of pluripotency, chemo-radioresistance/apoptosis, and targets of mRNAs were analyzed by real-time reverse transcription-PCR (qRT-PCR). Radiosensitivity was analyzed using a clonogenic assay after irradiation. Response to cisplatin, 5-FU, doxorubicin, and paclitaxel was done with MTT assay. Statistically significant decrease of K-RAS (*p* = 0.0369) and CASPASE3 (*p* = 0.0342) were observed with the growing expression level of let-7d. Cisplatin, 5-FU and doxorubicin caused similar decreased of cell survival with the increase of let-7d level (*p* = 0.004, post-trend *p* = 0.046; *p* = 0.004, post trend *p* = 0.0005 and *p*<0.0001, post trend *p* = 0.0001, respectively). All models were resistant to paclitaxel, irrespective of let-7d expression levels. Only two of the generated models (A and C) were radiosensitive (*p* = 0.0002). Conclusion: the above results indicated that the level of let-7d expression is an important factor for cell response to irradiation and chemotherapeutics.

## Introduction

Hypopharynx cancer is an uncommon cancer of the head and neck area. It occurs both in males and females, primarily aged between 50 and 60 years. In most cases, hypopharyngeal cancer is of a mucosal squamous cell origin, preceded by various precancerous lesions. Hypopharyngeal cancer does not usually have early symptoms, along with a high ability to metastasize, thus causing the lowest survival rate of all head and neck cancers. The treatment approach is based on surgery and postoperative radiotherapy. These advanced, unresectable cancers are treated by radiation and/or chemotherapy. Personalization of therapy might improve patients’ survival, but it is necessary to find biomarkers describing radio- and chemosensitivity for this kind of tumor. One promising group of biomarkers is miRNAs.

In addition, miRNAs are about 22 nucleotides long, with non-coding RNAs being well-known [1]. The miRNA precursors are transcribed from the genome (pri-miRNA) and create immature forms of miRNA called pre-miRNA—which are transformed to about 22 nt duplexes by Drosha and Dicer enzymes. One of the duplex strands, the guide strand, was incorporated into the RISC complex, which usually binds to 3’UTR region of target mRNA [2–4]. miRNAs can regulate from 30–60% of human genes [5]. Some data suggest that second strand, miRNA*, takes part in the regulation of gene expression [6]. These small RNAs are associated with cell cycle, apoptosis, proliferation, differentiation, metabolic pathways, and cell response to various stressors [7–12]. Half of the known miRNA genes are located close to or inside chromosome regions, usually mutated in cancer [13,14], which are fragile, unstable, and have cancer-associated sites. Single miRNAs function as tumor suppressors, oncogenes (oncomiRs) or has dual functions [15–20].

Lethal-7d (let-7d) is a member of the let-7 family, playing a critical role in the regulation of development and carcinogenesis. This family is conserved across species and is considered to be ancient [21]. Let-7 miRNAs are the most abundant among all miRNAs [22]. The let-7 family is involved in many cellular processes, having an important impact in cancer formation, progression, and metastasis. The family acts as tumor suppressor miRNA, and regulates expression of many oncogenes by both direct and indirect pathways [23–27]. The overall expression of let-7 family members decreases in human cancers and stem cells, but also have high expression levels in some cases [28]. The expression of let-7 is deregulated in cancers such as: pancreatic, prostate, primary pigmented nodular adrenal dysplasia, head and neck, ovarian, breast, bladder, kidney, and retinoblastoma [29–37]. The implication of the let-7 family in cancer is multifaceted; it regulates cell-cycle, apoptosis pathways, chemo- and radiosensitivity of the cell, as well as influences on tumor-initiating cells (TICs) formation and epithelial-to-mesenchymal transition process (EMT) [38–41].

This study is based on our observation that in some cases of head and neck squamous cell carcinoma (HNSCC), let-7d is upregulated in tumor tissue [42]. In this work, we tried to answer the question whether different levels of let-7d expression influence on sensitivity to chemo- and radiotherapy.

## Methods

### Cell line culture

The FaDu cell line and its variants were maintained in Dulbecco's Modified Eagle Medium (DMEM) with high glucose (4.5 g/L, Sigma), supplemented with 8.85% (v/v) of fetal bovine serum (Sigma), 1.77 mM L-glutamine (PAA), 0.885% (v/v) MEM non-essential amino acid solution (PAA), 0.885% (v/v) penicillin-streptomicin (PAA), and 8.85 mM HEPES (Sigma). The 293T cell line used for the production of lentiviral vectors was cultured in DMEM with high glucose (4.5 g/L) medium (Sigma) supplemented with 8.85% (v/v) fetal bovine serum (Sigma) and 20 mg gentamicin (Kirka). Cell lines were cultivated in a humidified 5% CO_2_ atmosphere at 37°C. The mycoplasma detection tests (Minerva) were performed routinely during cell line culturing. All cells used in the experiments were the same ones used in the 15^th^ splitting.

### Constructs designing, plasmids production, and lentiviral transduction

The let-7d gene sequence and its flanking sequences (about 180 nucleotides) were amplified by the PCR method using two pairs of primers: F1: 5’CAATTTAAATGTCATATGGCCAGATA and R1: 5’CATTAATTAAAGTTATCAATGTCAGCA; F2: 5’TCACTCGAGGTCATATGGCCAGATA, and R2: 5’TCACTCGAGAGTTATCAATGTCAGCA (two different pairs of primers to achieve single and double let-7d cassettes). Purified inserts were cloned into pWPXL-GFP plasmids between PacI and SwaI sites. The obtained plasmids were sequenced to verify the correctness of amplified inserts. Lentiviral particles were generated by cotransfecting 20 μg of lentiviral vectors (pWPXL-7d, pWPXL-7d-7d, pWPXL-GFP), 15 μg of p89.1 and 7 μg of pMD2G of packing vectors in 293T cells using the CaCl_2_/HBS method. FaDu-7d, FaDu-7d-7d, and FaDu-GFP cell line models were achieved through lentiviral infection at the single and multiple infection sites (maximum 3 times). The clonal selection was performed.

### Characterization of cell line models

#### The miRNA expression levels

The total RNA from cell line models with different levels of let-7d expression was isolated using the TRI Reagent (Sigma). The miRNA levels were defined by a two-step qRT-PCR method, using TaqMan microRNA Assay (Applied Biosystems, Foster City, CA, USA). Data obtained were compared to FaDu-GFP cell lines and calculated with the 2^-ΔΔCt^ method. The snoRNA U18 was used as an internal control.

#### Proliferation

The proliferation ratio of cell line models was measured by 3-H thymidine incorporation assay. 7000 cells per well in 96-well dishes were seeded. After 48 h of incubation, the 3-H thymidine was added. Incorporation into DNA was measured with a scintillation counter (MicroBeta PerkinElmer, Waltham, MA, USA) and compared to the FaDu-GFP cell line.

#### The qRT-PCR of selected genes

The qRT-PCR primers for genes involving pluripotency (OCT3/4, SOX2, NANOG), chemo-, radio-resistance/apoptosis (ATM, ABCB1, BAX, CASPASE3, BCL2) and let-7d targets (DICER, HMGA1/2, MYC, H-RAS, K-RAS, N-RAS, ARID3A) were designed with the Universal ProbeLibrary Assay Design Center (Roche Applied Science, Basel, CH). The reverse transcription PCR was performed using the iScript cDNA synthesis kit (Bio-Rad, Berkeley, CA, USA) while the qRT-PCR was performed with the 2x SYBR Green master mix (Roche, Basel, CH) and Light Cycler 96. Results were calculated with the 2^-ΔCt^ method. The GAPDH gene was used as a reference, and melting curves discriminated non-specific products of PCR reactions. Each experiment was repeated at least 3 times.

### Response to irradiation

Cell line irradiation experiments were performed by the Medical Physics Department. Cells were irradiated on a Varian Clinac 2300 linear accelerator. The cells were irradiated in a special water phantom MP1 (PTW, Freiburg, GY) with 6 MV accelerating potential.

#### Clonogenic assay

The cells were seeded in 25 cm^2^ culture flasks, which were filled up with PBS, and were irradiated using a dose of 2 Gy. Following that, irradiated and control (non-irradiated) cells were seeded in 6-well plates. The cells were cultivated for 10–14 days. Next, the colonies were stained and counted.

### Response to chemoexposure

#### IC_50_

The IC_50_ of cisplatin, 5-FU, doxorubicin, and paclitaxel for the FaDu cell line was defined using the MTT assay.

#### MTT assay

7000 cells per well of cell line models were seeded in 96-well plates. The *IC*_*50*_ concentration of drugs was added and incubated for 48 h with cells. The results for samples treated with the drug were compared to those without drug treatment, as well as being compared to control (FaDu-GFP) line.

### Statistical methods

Statistical analysis was performed with the use of Prism v. 4.0 (GraphPad Software, San Diego, CA, USA). All experimental values were obtained in at least three independent experiments and presented on average with standard deviation error bars. A two-tailed *p* < 0.05 was considered to represent a statistically significant result. The different types of variables (i.e., expression level of let-7d; expression levels of pluripotency, drug resistance biomarkers; proliferation; irradiation, and chemoexposure response) were measured in subsequent A-E cell lines with different levels of let-7d expression; these were analyzed using one-way analysis of variance (the ANOVA test) with either a post-test for trend or a comparison of each cell line to the control. The Bonferroni correction was used for estimation of post-test significance.

## Results

### Characteristics of the FaDu/let-7d cell line models

**Fig 1:**
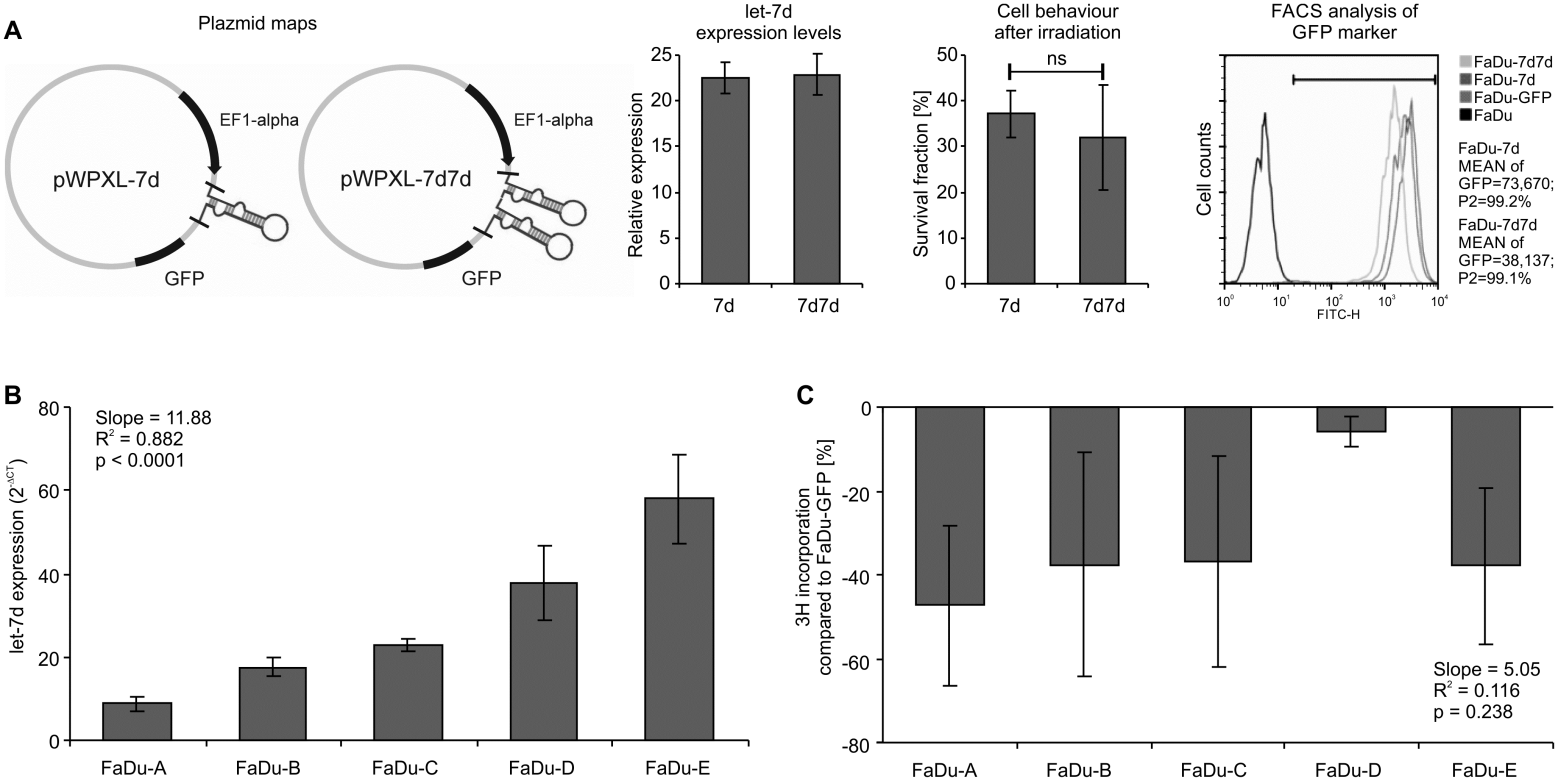
Characteristics of cell line models. A) The FaDu cell line has been transduced using two different lentiviral constructs, containing single and double cassettes of let-7d. The let-7d expression levels were similar, but the MEAN of GFP fluorescence marker was nearly half lower in the 7d7d line than in 7d. The irradiation effect in both cases was similar. B) Five cell line models based on the FaDu cell line have been stable for further experiments. The expression for each model was calculated, comparing it to the FaDu-GFP cell line, using the 2^-ΔΔCt^ method. The following results were found: A: 8.72; B: 17.44; C22.85; D:37.93, and E:57.89; C) the proliferation ratio generally decreased in the overexpressed let-7d cell line, compared to the controls for the FaDu-GFP line; however, differences between models were not statistically significant, and as such, we did not observe a trend.

Different levels of let-7d expression have been achieved using the lentiviral system. The modified cells were chosen using a clonal selection method, in order to achieve a cell line with a “mother” genetic background. The main limitation of this experimental approach is the unpredictable position of the lentiviral integration: this may affect the expression of neighboring genes. Nevertheless, this system is considered safe and is commonly used for RNAi experiments, as well as for induced pluripotent stem cell (IPS) production. To verify that the side-effects of lentivirus did not affect cell response, we compared cells with single and double cassettes of let-7d. Two FaDu cell lines were created with a single cassette of let-7d (FaDu-7d) and with a double cassette of let-7d (FaDu-7d7d). The second one was generated with the use of half amount of lentivirus with the double let-7d construct for transfection. The FACS analysis of GFP confirmed that FaDu-7d7d contained twice less amount of virus particles. The expression level of let-7d in the both lines was the same, what was reflected with the same behavior after irradiation, (Fig 1A). Experimental results allowed us to conclude that observed effects were caused by let-7d.

Using the lentiviral system and the single clone selection method, six cell line models (A, B, C, D, E, and reference FaDu-GFP) were obtained. The expression level of let-7d for each model was calculated, compared to the FaDu-GFP cell line. The following results were obtained: A: 8.72; B: 17.44; C: 22.85; D:37.93, and E: 57.89. The flow cytometry analysis of the GFP marker showed high homogeneity (over 97% of FITC-H) of positive cells compared to non-transduced FaDu cells (Fig 1A). Models differed from each other by the expression level of let-7d, such that these differences were statistically significant (*p* < 0.0001). Models were ordered from A with the lowest level of let-7d to E as the highest one; in this composition of let-7d expression, a linear trend was shown (*p* < 0.0001) in Fig 1B. The proliferation ratio generally decreased in overexpressed let-7d cell lines, compared to the control FaDu-GFP line (Fig 1C), but differences between models were not statistically significant (*p* = 0.3739) and a trend in groups was not observed (*p* = 0.2379). Although model D seemed to behave differently from the others the difference is not statistically significant and therefore the most likely it does not result from biological features.

### Influence of different levels of let-7d on expression of selected genes

**Fig 2:**
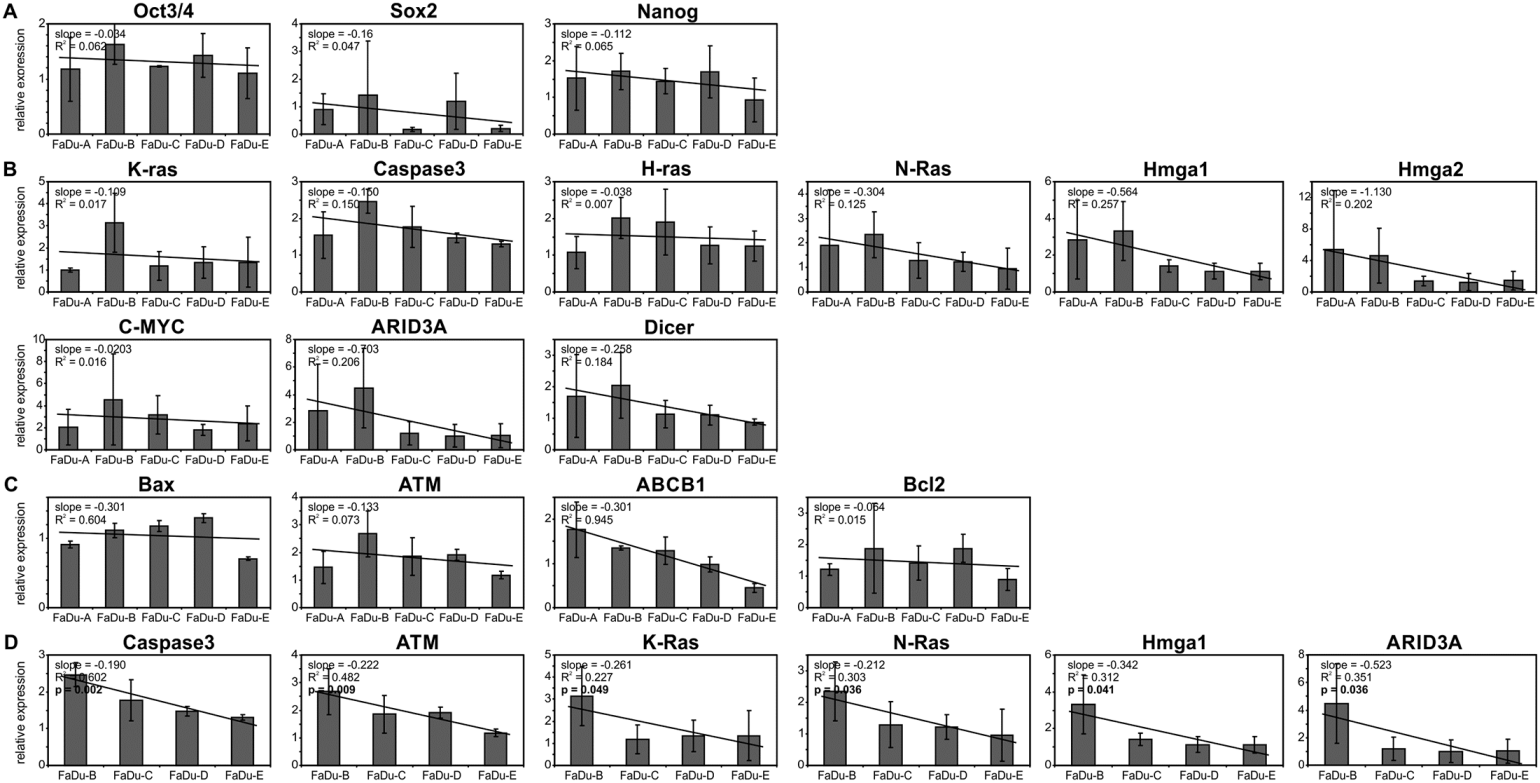
Expression of genes characteristic for. A) pluripotency (OCT3/4, SOX2, NANOG) and B) let-7d targets: K-RAS, Caspase3, H-RAS, N-RAS, HMGA1, HMGA2, C-MYC, ARIDA3A, DICER; C) genes connected with chemo-radioresistance/apoptosis (BAX, ATM, ABCB1, BCL2); D) statistical analysis of models B-E indicated positive results for mRNAs: Caspase3, ATM, K-RAS, N-RAS, HMGA1, and ARID3A.

There were no differences among models for OCT3/4, SOX2 and NANOG mRNAs expression (Fig 2A). Analysis of let-7d mRNA targets revealed statistically significant differences in expression among models for K-RAS (*p* = 0.0369), without a trend and for CASPASE3 (*p* = 0.0342; post-trend *p* = 0.0675). Other let-7d targets mRNA did not show statistically significant differences (Fig 2B). In the group of analyzed mRNAs connected with chemo-radioresistance/apoptosis, differences in the expression among models, we observed for BAX *p* = 0.0111 (but not trend *p* = 0.344) and ATM *p* = 0.0506 (but not trend *p* = 0.203). For ABCB1 expression, differences among models were not statistically significant (*p* = 0.2029) but a decreasing trend of expression was observed in the group of models (*p* = 0.034). Expression level of BCL2 did not differ among the analyzed models. This part of the analysis was presented in Fig 2C. Summarized results showed that model B differed from the others, due to overexpression in nearly all studied mRNAs. Model E demonstrated the lowest expression of all analyzed mRNAs. This can exclude model A from statistical analysis. Models B-E showed statistically significant differences among the models for CASPASE3 (*p* = 0.01), ATM (*p* = 0.044), and positive trends for most analyzed mRNAs: CASPASE3 (*p* = 0.0017), ATM (*p* = 0.0090), K-RAS (*p* = 0.0497), N-RAS (*p* = 0.0364), HMGA1 (*p* = 0.0414), and ARID3A (*p* = 0.0367). It must be noted that although expression of most genes does not show a significant trend (due to high levels between-model variation) when we observe almost all cases, the expression of analyzed genes decreases with increasing levels of let-7d (value of the slope and correlation coefficient R is almost always < 0). We expected that increased expression of let-7d (A-E) would show decreased expression of mRNAs. This indicates the expected let-7d’s expression of all studied genes; however, the effect was too small, while the experimental variety was too large, yielding no statistical significance for the individual gene. Data is presented in Fig 2D.

### Response of FaDu models to cisplatin, 5-FU, paclitaxel, and doxorubicin

**Fig 3:**
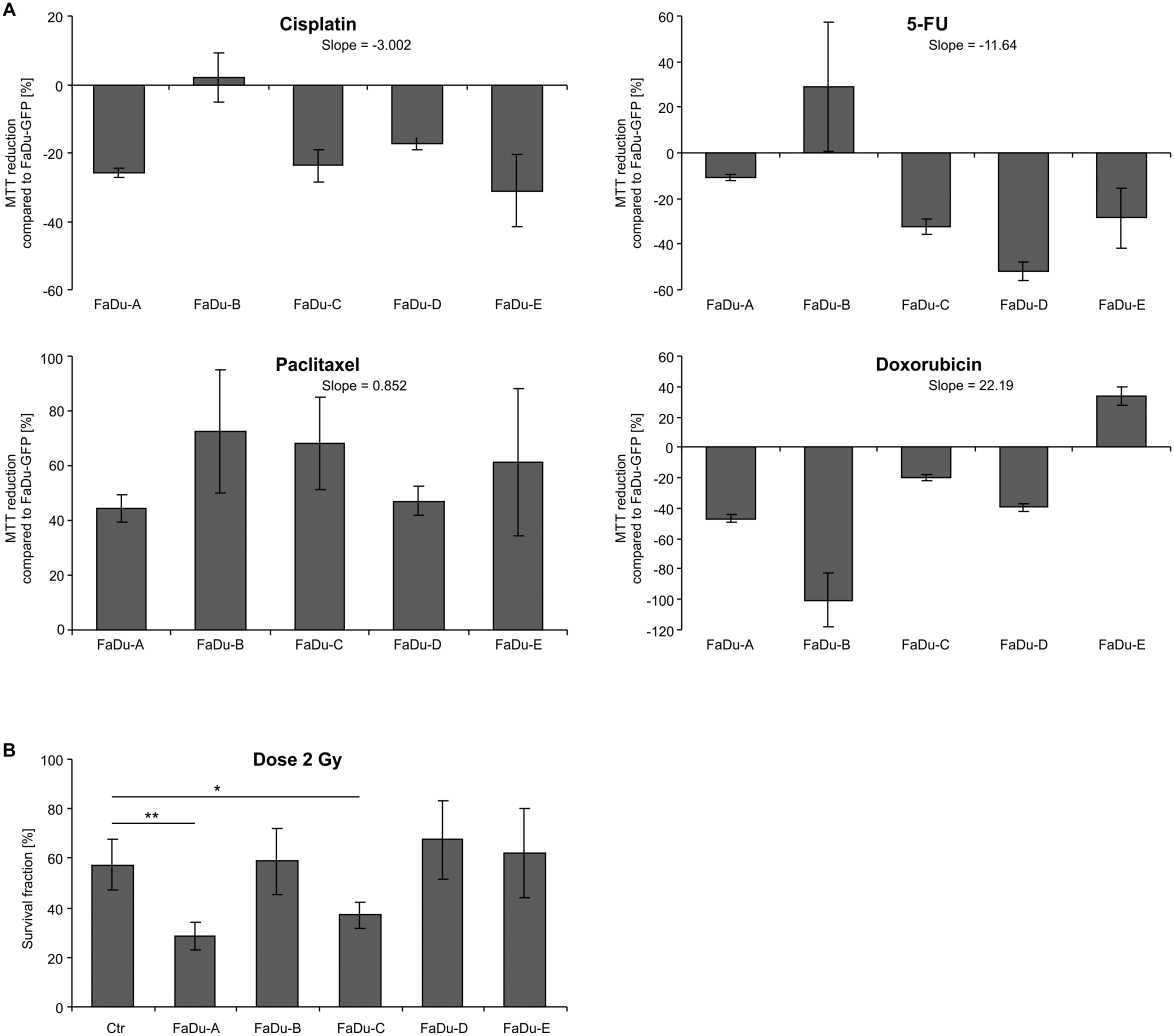
Response of FaDu let-7d models to chemo-and radiotherapy. A) The cell line models (A-E) were exposed to chemotherapeutics: cisplatin (1.12 μg/mL); 5-FU (0.86 μg/mL); paclitaxel (0.54 μM), and doxorubicin (0.06 μM) were compared to FaDu-GFP B) Survival fractions [SF%] of the cell models were assessed according to a dose of 2 Gy. The differences in survival were statistical significant (*p* = 0.0002) for the models A and C.

The cell line models (A-E) were exposed to chemotherapeutics used in HNSCC treatment. The IC_50_ dose was established using the non-modified FaDu cell line and was as follows: 1.12 μg/mL for cisplatin; 0.86 μg/mL for 5-FU; 0.54 μM for paclitaxel, and 0.06 μM for doxorubicin. Cisplatin, 5-FU and doxorubicin caused a reduction of the proliferation ratio in all models. Cisplatin decreased cell survival by about 20% compared to control in FaDu-GFP (with the exception of model B). The difference in cell survival among models was statistically significant (*p* = 0.0014) and a linear trend was also observed (*p* = 0.0460). We noted that 5-FU also caused a decrease of cell survival (except in model B). Differences in cell survival among models were statistically significant (*p* = 0.004) and the linear trend was present (*p* = 0.0005). Similar results were obtained for doxorubicin, which generally caused reduction of cell survival (except in model E). Differences among models were statistically significant (*p* < 0.0001), as we observed a linear trend (*p* < 0.0001). Models B and E demonstrated a different response to chemotherapeutics. Model B was resistant to cisplatin and 5-FU. Model E was resistant to Doxorubicin. Contrary to the above chemotherapeutics, paclitaxel caused about 40% higher cell survival in comparison to the control in FaDu-GFP, without differences among models (*p* = 0.1484) or linear trends (*p* = 0.7651). All models were resistant to paclitaxel irrespective of let-7d expression level. Experimental results are summarized in Fig 3A.

### Response of FaDu models to irradiation (IR)

The cell line models were irradiated by a dose of 2 Gy, culturing 10–14 days, at which point the survival fraction (SF) was calculated. The results for each model were as follows: A—30.71%; B—54.33%; C—36.67%; D—71.36%; E—59.81%, and FaDu-GFP—60.08%. The differences in survival fraction were statistically significant (*p* = 0.0002) only for models A and C. The linear trend was not observed. Experimental data is summarized in Fig 3B.

## Discussion

The role of let-7 in cancer development and diagnostics has already been described. Currently, it is considered to be a therapeutic application, based on the restoration of the normal let-7 level. This approach is recommended for those particular cancers, which show underexpression of miRNA. On the other hand, let-7 may be also be overexpressed, which indicates that let-7 does not play an only suppressor function, but can also act as oncomiR. This notion, among others, was supported by experiments on liver cells showing that chronic overexpression of let-7 caused liver damage, degeneration, and finally cancer [43]. Overexpression of let-7 may be induced by different mechanisms: from regulation of let-7 cluster transcription via changes in maturation machinery to global deregulation of cell pathways. Firstly, the effect of overexpressed let-7d was showed by Chang et al [44] demonstrating that let-7d negatively modulated TWIST and SNAIL expression. However, target mRNA analysis in this work revealed statistically significant result only for K-RAS and CASPASE3. It may be assumed that a lack of substantial changes in expression levels of studied mRNAs was caused by high levels between-model variation. On the other hand, in most cases, we observed a negative or close to 0 slope, which suggested that different let-7d levels inhibited expression of the examined mRNAs. Another explanation of our results relates to the very low level of let-7d expression in FaDu cells, in fact, generated expression of let-7d in the models was still too low to see substantial changes in analyzed mRNA expression. This notion is in line with the observation that when model A was excluded, a statistically significant trend was observed for CASPASE3, ATM, K-RAS, N-RAS, HMGA1, and ARID3A. This may suggest that more noticeable differences for the investigated genes expression may be observed for higher levels of let-7d than presented in model A and the effect of let-7d overexpression ought to be analyzed in models with its expression higher than in model E. Thirdly, let-7d belongs to the family including 10–13 members [27], sharing common mRNAs targets but having their own specific characteristics. Some authors postulate that the overall level of the let-7 family is important for its biological effect [45]. We analyzed the influence of let-7d expression, but it is possible that not all of its levels harmonized with those of other members of the family. The specific FaDu genetic context influenced our data.

### Cell response to chemo- and radioexposure

The main question of this study is how different expression levels of let-7d affect sensitivity to chemo- and radiotherapy. Tumor resistance to chemotherapeutic drugs is the result of deregulation of various cell mechanisms, such that finding the biomarker of chemosensitivity for this group of drugs is a real challenge. On the other hand, loss of rerelet-7 family, in particular, has been correlated with drug resistance in many tumor types, as well as maintenance of an appropriate expression level (correlated to successful chemotherapy). In the case of patients with esophageal cancer, let-7b and let-7c were used as indicators of sensitivity to cisplatin [46]. Let-7 affected esophageal squamous cells by regulating IL-6/STAT3 pathway so that the designation of let-7b/c expression level was useful in treatment choice [46]. The influence of overexpressed let-7d on chemoresistance has been less examined. In the above-cited work [44], authors observed that overexpressed let-7d inhibited chemoresistance to cisplatin and paclitaxel in OSCC-ALDH1+ cells; this observation was correlated with downregulation of the multidrug resistance (MDR) gene. Our experiments showed that with the exception of model B increasing expression of let-7d has influenced on response to cisplatin (with a linear trend). Additionally, a decreasing trend of ABCB1 expression was observed. Similar results were obtained for 5-FU and doxorubicin. Model B was resistant to both Cisplatin and 5-FU, whereas model E was resistant to Doxorubicin, which suggested that despite the noticed linear trend, not each overexpression level of let-7d was sensitized to chemotherapeutics. To explain in more details above results we analyzed expression levels of targets/apoptosis/chemoresistance genes but the list of mRNAs showing statistically significant differences among models in expression levels was very short (CASPASE3, BAX, and ATM, slop for ABCB1). In subgroup containing models B-E decreased expression level was noticed for CASPASE3, ATM, and HMGA1. Involvement of these molecules in chemo-resistant/sensitivity was documented. It is possible that let-7d influences sensitivity to chemotherapeutics both by regulating (direct or indirect) of the genes connecting with apoptosis and by repressing the genes promoting self-renewal/TIC formation. Based on this limited number of analyzed mRNAs our explanation has speculative character. FaDu is described as an aggressive cell line, therefore, its transcriptome needs for explanation real influence of let-7d as well as experiment with paclitaxel. Paclitaxel caused a nearly 40% higher cell survival compared to the control arm of FaDu, which did not depend on let-7d level. This might be connected with specific FaDu cells in genetic disorders.

In experiments with irradiation, statistically significant differences in survival fraction were obtained only for models A and C. Generally, let-7 family is overexpressed in radiosensitive cancer cells with the exception of let-7g, which overexpression was associated with radioresistance. It was also postulated that let-7 radiosensitized cells via regulation of the RAS family [47, 48]. This idea was experimentally confirmed by Oh et al regarding human pancreatic (ASPC1) and lung tumor (A549) cell lines [48]. The authors demonstrated that overexpression of let-7a led to downregulation of K-RAS, creating radiosensitivity of investigated cell lines. Even though the higher let-7 expression is connected to a radiosensitive phenotype after irradiation, let-7 expression decreased [49]. The mechanism of this process is not clear, although it probably involved other molecules controlling cellular response to IR (in the case of let-7a and let-7b, in which expression was reduced by wild type p53) [49]. Looking for radiosensitivity in the miRNA signature for HNSCC patients, Liu et al [50] validated data obtained from in vitro experiments by analyzing patient samples in the TCGA base. Patients with a complete response were characterized by upregulation of miR-16, miR-29b, miR-150, miR-1254, and down-regulation of let-7e after irradiation. Additionally, ATM expression levels were lower, compared to those in resistant patients. In our experiments, only two models responded to irradiation, but we did not find an explanation about why this phenomenon took place. It should be indicated that the FaDu cell line is known to have mutations in CDKN2A (c.151-1G>T), SMAD4 (c.1 1659del1659), TP53 (c376-1G>A; c.743G>T) genes. It is possible that changes in p53 caused that effect of certain levels of the let-7d effect to be more visible. However, we concluded that the level of the let-7d expression affected the IR response.

## Conclusion

The determination of individual miRNA expression changes can be an important marker of successful patient treatment. We focused on let-7d, known as miRNA suppressor molecule, for which overexpression was described in HNSCC. In other studies both over and underexpression of let-7d in different cancer types were also observed. In fact, the actual level of let-7d differ substantially between cancer samples and cannot be simply classified either as a high (overexpression) or low (underexpression). Therefore in our study, we focused on the question, whether incrementally different levels of let-7d influence chemo- and radiosensitivity. Generally, increased levels of let-7d enhanced cells sensitivity to chemotherapeutics. Only two of the investigated models showed an effect after irradiation. On the other hand, growing level of let-7d expression caused a declining tendency in the expression of the studied genes but it does not let to connect these specific radio- and chemoresistant let-7d levels with changes of expression of investigated mRNAs. Summarizing, we found that level of let-7d was of significance for cell response to irradiation and chemotherapeutics.
